# Selected discoveries from human research in space that are relevant to human health on Earth

**DOI:** 10.1038/s41526-020-0095-y

**Published:** 2020-02-12

**Authors:** Mark Shelhamer, Jacob Bloomberg, Adrian LeBlanc, G. Kim Prisk, Jean Sibonga, Scott M. Smith, Sara R. Zwart, Peter Norsk

**Affiliations:** 1 Johns Hopkins University School of Medicine, Baltimore, MD USA; 2 NASA Johnson Space Center, Houston, TX USA; 3 Baylor College of Medicine, Houston, TX USA; 4 University of California, San Diego, CA USA; 5 University of Texas Medical Branch, Galveston, TX USA

**Keywords:** Translational research, Research management

## Abstract

A substantial amount of life-sciences research has been performed in space since the beginning of human spaceflight. Investigations into bone loss, for example, are well known; other areas, such as neurovestibular function, were expected to be problematic even before humans ventured into space. Much of this research has been *applied* research, with a primary goal of maintaining the health and performance of astronauts in space, as opposed to research to obtain fundamental understanding or to translate to medical care on Earth. Some people—scientists and concerned citizens—have questioned the broader scientific value of this research, with the claim that the only reason to perform human research in space is to keep humans healthy in space. Here, we present examples that demonstrate that, although this research was focused on applied goals for spaceflight participants, the results of these studies are of fundamental scientific and biomedical importance. We will focus on results from bone physiology, cardiovascular and pulmonary systems, and neurovestibular studies. In these cases, findings from spaceflight research have provided a foundation for enhancing healthcare terrestrially and have increased our knowledge of basic physiological processes.

## Introduction

For as long as humans have ventured into space, research has been performed to study the physiological effects of spaceflight. The claim is sometimes made that the only reason to do this research on humans in space is to provide the information that is needed to keep them alive and well when they are in space—the implication being that there is little or no fundamental scientific return from such research.^1^ Here, we provide evidence to the contrary. In the course of applied research that is primarily focused on maintaining astronaut health in space, discoveries have been made that have a broader impact on fundamental scientific understanding. That these findings arose in the course of applied research suggests that there will be other interesting and important scientific discoveries when and if more open-ended exploratory science can be performed in space. We present here five areas of physiology that support this contention.

## Bone Loss in Space: Countermeasures for Disuse

Even in the years prior to human spaceflight, clinical research suggested that weightlessness would lead to bone loss, although the details were uncertain.^2,3^ Ongoing NASA research has provided a more general understanding of bone loss and delineation of the detailed effects of disuse on bone, independent of disease-related bone loss and recovery. The early Skylab flights and short-duration missions of Gemini and Apollo led to the development of densitometry techniques that are now used widely in clinical settings around the world.^4,5^ This research shed light on the rapid bone loss caused by disuse conditions.

Spaceflight research found that bone loss could be continuous for several months in space, and suggested that recovery after flight would not be rapid, if it occurred at all. Measurements on the international crews of Mir, Shuttle/Mir, and ISS documented continuous loss of areal bone mineral density of 1–2% per month, which varied greatly between individuals, bones, bone compartments, and skeletal regions within the same individual.^6–8^ These studies also showed the dichotomy between cortical and trabecular bone loss with disuse. Some recovery of lost bone does occur very slowly following return to Earth, but some loss within the trabecular compartment may be permanent.^8–10^ This information has been important for NASA planning and also for terrestrial clinicians attempting to rehabilitate patients after significant periods of disuse. Mechanistically, during disuse, systemic markers of bone resorption and urinary calcium excretion are consistently elevated, while bone formation is unchanged, suggesting that remodeling is elevated and the dynamics are uncoupled.^11^ Alterations in remodeling have been well documented in most bone diseases, and now it is known that spaceflight and disuse can result in altered remodeling in *healthy* subjects, hence the need to prioritize procedures to minimize the effects of disuse to the greatest extent possible. NASA has investigated exercise countermeasures, such as bicycle and treadmill ergometry and resistive weight training, that appear to be partially effective.^12^ In addition to routine exercise, an antiresorptive agent, alendronate, taken weekly, has proved to be effective in reducing bone loss, remodeling rate, and urinary calcium excretion.^13^ These results had been verified using the ground-based bed-rest model of weightlessness, without exercise.^14^

Notwithstanding the obvious benefits of this research for science and astronaut health, these findings may also be important for disuse-related bone changes on Earth. For example, amputation, lower-leg fractures, ligament tears, and other clinically-required but temporary disuse conditions lead to bone loss with delayed or incomplete recovery.^15,16^ The value of an antiresorptive medication in these surgical situations requires future validation. The spaceflight studies indicate that an early pharmacologic intervention at the beginning of a disuse episode could be effective in preventing bone loss and, thereby, preserve bone integrity (especially trabecular structure critical to bone strength).

## Bone Loss and Nutrition: Calcium Metabolism and Dietary Proteins

Nutrition is critical for human health, especially during exploration in remote regions, where food and nutrient intake may be compromised. Spaceflight provides a highly controlled environment with healthy individuals and a high degree of monitoring. Therefore, it is an ideal experimental setting in which to analyze the association of nutrients with rapid bone loss and to test the effects of specific nutrient supplementation. This has led to a better understanding of the effects of dietary components on bone,^17^ and of the effectiveness of vitamin D supplementation in individuals without natural ultraviolet light exposure.^18^

Iron stores (reflected by serum ferritin) increase in the early weeks of spaceflight, then slowly return toward pre-flight levels.^19^ The increase is associated with evidence of oxidative damage and bone resorption. Greater extent or duration of ferritin increase during spaceflight results in a greater decrease in bone mineral density (hip, trochanter, hip neck, pelvis) after long-duration flight.^20^ Also, the ferritin increase has been shown to correlate with elevated biochemical markers of bone resorption and urinary calcium excretion during spaceflight. Several human and animal studies support these findings and show that mild iron excess (but still within a normal clinical range) is associated with bone loss by a mechanism believed to be related to oxidative stress.^19,21^ The relationships between iron and bone loss during flight were observed in 4–6-month missions; these same relationships in a terrestrial study were observed after 3 years,^21^ likely due to the fact that bone loss during spaceflight is approximately ten times faster than on Earth. These findings help identify relationships between nutrition, oxidative stress, and bone loss and also have implications for terrestrial health and medicine.

Other dietary factors can influence acid/base balance in the body, which in turn can affect the skeleton, a source of base (calcium carbonate) that can neutralize acid loads. Dietary proteins can contribute to acid load through the production of low levels of sulfuric acid. Therefore, a high dietary ratio of animal protein (rich in amino acids) to potassium (typically as base-producing, acid-neutralizing salts) is associated with increased bone breakdown.^20^ Furthermore, supplementing a nominal diet with amino acids during extended bed rest (an analog of spaceflight) leads to increased acid load and increased bone resorption.^22^ Recent spaceflight research has identified that the acid:base potential of the diet is negatively correlated with bone loss (more acidogenic, more bone loss).^17^

The effect of diet on bone loss remains controversial—some studies have shown protective effects of a high protein diet, but some studies found a high protein diet could be detrimental. These discrepancies are likely due to other contributing factors, such as calcium intake.^23–25^ As with almost any human research, controlling for all relevant variables and confounding factors is challenging. For example, postmenopausal women often suffer from decreased bone density, but these individuals have varying diets, levels of physical activity, comorbidities and genetic influences. Further, it can take years to observe any therapeutic or detrimental effects of diet since bone loss is much slower than in space. Astronaut populations are relatively homogenous and free of comorbidities yet experience accelerated bone resorption and consume foods from the same controlled and limited food system, with a shared key environmental stressor: microgravity. Therefore, the space environment holds the potential to provide insights into the therapeutic or detrimental effects of nutrients on bone loss that can be applied to clinical research.

## Cardiovascular System: Lessons Learned About Central Venous Pressure and Left Ventricular End-Diastolic Volume

Blood and other fluids shift from the lower extremities to the upper body during spaceflight due to the absence of a hydrostatic gradient in weightlessness.^26^ It has been speculated that the repositioning of fluids would increase cardiac preload and central venous pressure (CVP), with implications for cardiac function. (Preload, or end-diastolic volume, is the amount of blood in the ventricles at the end of cardiac filling, before the systolic “pulse” that sends blood to the peripheral circulation. Higher preload increases the volume of blood flow in the subsequent contraction.) CVP in space was first measured with catheters in arm (cubital) veins and was unexpectedly decreased in flight.^27–29^ In another study, CVP was more directly measured with central fluid-filled catheters during the initial hours of two Space Shuttle missions. These and other measurements confirmed an in-flight reduction in CVP and also found an increase in left ventricular end-diastolic volume (LVEDV, the amount of blood in the ventricle at the end of filling, just before the systolic pulse).^30,31^ It was surprising to find decreased CVP yet increased LVEDV because it is venous pressure (CVP) that provides the force to fill the ventricles (LVEDV). Decreased CVP reflects a decrease in cardiac preload and, thus, should result in a decrease in subsequent atrial volume and LVEDV.

The ostensible contradiction of a decrease in CVP and an increase in LVEDV^30^ was later explained by data from the weightless phase of parabolic flight, where a decrease in CVP of 1.3 mm Hg was observed.^32^ At the same time, esophageal pressure, which reflects intrathoracic pressure (inside the chest cavity, surrounding the heart), fell by 5.6 mm Hg. The fact that esophageal pressure fell more than CVP provided the key to understanding the simultaneous decrease in CVP and increase in LVEDV in weightlessness. While supine in 1 g, the thorax is mechanically compressed, which increases CVP relative to ambient pressure. The importance and magnitude of mechanical compression pressure for understanding preload changes to the heart was not known before CVP was measured in weightlessness.^32^

The relationship between the expansion of the thorax and stroke volume and cardiac output observed during spaceflight has contributed to an understanding of the effects of posture on heart-lung interactions. The weightlessness-induced change in CVP relative to intrathoracic pressure (transmural CVP) during spaceflight leads to increases in stroke volume and cardiac output of 35% and 41%, respectively, compared to seated upright in 1 g,^33^ in accordance with the well-known Starling effect^34^ which states that stroke volume increases in response to increases in blood volume in the ventricles prior to contraction. Understanding that expansion of the thorax helps to increase stroke volume and cardiac output contributes to basic knowledge of the influence of gravity and posture on heart-lung interactions. As an example, measuring CVP as an indication of the change in cardiac preload during a change in posture (from supine to head-up or vice versa) can lead to erroneous interpretations of the measurements, if intrathoracic pressure is not taken into account. The front-to-back compression of the thorax while supine increases intrathoracic pressure, which leads to increased LVEDV despite decreased CVP. The same is likely also true when shifting from supine to horizontal lateral positions because of a different distribution of gravitational loading on the thorax.

The findings from space and parabolic flights are also relevant for understanding how intrathoracic pressure regulation can aid in the treatment of hypotension and in decreasing intracranial pressure. Use of an inspiratory threshold device (ITD), which increases resistance to inspiration, can increase cardiac filling via negative-pressure-induced expansion of the thorax during inspiration, resulting in the suction of more blood into the right atrium and ventricle.^35^ The ITD can also decrease intracranial pressure in patients with intracranial hypertension by increasing venous return and decreasing cerebrospinal fluid pressure through the inspiratory expansion of the thorax. These medical advances would not have been possible without the measurements from spaceflight, parabolic flight, and bed-rest research.

## Pulmonary Function: Gravity-Induced Lung Deformation

Gas exchange in the lung is accomplished by passive diffusion, in which inspired gas and circulating blood must be brought together in appropriate proportions across a very thin (alveolar) membrane. In the 1950s, after the advent of radiolabeled tracers, the effects of gravity on the perfusion of the lung were first appreciated. The first few studies found that ventilation varied according to gravity level and, together with very low pulmonary vascular perfusion pressures, affected gas exchange, with a gravity-induced reduction in exchange efficiency as a direct consequence of ventilation-perfusion mismatch.^36–39^ These observations help to explain much of the age-related impairment in pulmonary gas exchange as the aging lung in geriatric patients deforms more under the influence of gravity.

The expectation then was that in weightlessness ventilation, perfusion, and the matching of ventilation to perfusion would become uniform (lacking in gradients due to gravity).^40^ Observations showed that while ventilation (the air that reaches the alveoli)^41^ and perfusion (the blood that reaches the alveoli)^42^ were indeed much more uniform, some residual inhomogeneity remained, likely due to the complicated structure of the lung. The observations showed, more importantly, that the matching of ventilation to perfusion was no better than that seen on the ground.^43^ This initially paradoxical observation, which could not be readily appreciated in terrestrial experiments, is now known to be a direct consequence of the coupling of ventilation and perfusion. Gravity deforms the lung, resulting in uneven ventilation,^44^ and similarly causes uneven perfusion.^45^ Thus, in the normal human lung, gravity serves effectively to maintain the matching of ventilation to perfusion through mechanical effects^46^ and thus to maintain efficient gas exchange. However, as lung weight is increased – as is often the case in intensive-care situations with excess fluid accumulation – lung deformation increases, regions of the lung become unventilated, and gas exchange becomes compromised. Understanding the nature of lung deformation and how gravity affects gas exchange – an understanding improved through spaceflight studies— is a matter of critical importance in the intensive care unit where prone positioning in some patients results in significant reductions in mortality.^47^

## Sensorimotor and Neurovestibular Function: Perception and Orientation in Altered Gravity

Among the most widespread biomedical impacts from spaceflight may be those in the area of neurovestibular function, given its long history going back to high-performance aircraft flight, predating spaceflight.^48^ Research in this area has contributed to basic understanding of the remarkable capacity for adaptive plasticity. Humans have a lifetime of experience and neural development (and many years of evolution) in a constant gravity field. The combination of sensory information that results from movement in this gravity field takes gravity into account. Consider a head tilt: the vestibular semicircular canals sense angular velocity, the vestibular otolith organs detect a change in orientation with respect to gravity, and proprioceptors are triggered by neck flexion. In a weightless environment, the otolith tilt signal is missing, but despite a lifetime of experience with normal Earth gravity, astronauts adjust to this “missing” otolith signal within a matter of days.^49^ Aspects of this neuroplasticity have informed training programs to improve motor function in clinical populations. An example is a better understanding of the role of gravity loading in maintaining muscle and motor function, and the importance of maintaining loading to prevent degenerative changes in protein metabolism and muscle fibers that can impair rehabilitation after spinal-cord injury.^50^

Another unexpected observation was the ability to evoke caloric nystagmus in orbital flight.^51^ Nystagmus is the repetitive reflexive motion made by the eyes to maintain gaze stability when the head is rotated. It can also be stimulated, as a test of vestibular function, by irrigating the ear with hot or cold water, which sets up convection within the fluid of the semicircular canals. Since fluid in the canals under normal circumstances is only set in motion when the head moves, caloric stimulation mimics an extended head rotation and induces the corresponding nystagmus. The presence of caloric nystagmus in space was surprising because the dominant stimulus on Earth is convection in the fluid in the inner ear. With the weightlessness of space, there is no convection and therefore, there should be no caloric nystagmus. Later studies investigated this phenomenon and determined that there is a direct thermal effect of caloric stimulation on the semicircular canals of the vestibular system,^52^ which slightly expands the canal membrane and induces fluid motion within the canal. This furthered the understanding of this very commonly used diagnostic tool.

Another neurovestibular discovery that was uncovered during spaceflight research was the role of otolith asymmetry on ocular alignment and space motion sickness.^53^ The otolith organs in each vestibular labyrinth contain small crystals of calcium carbonate (otoconia); the relative motions of the otoconia detect linear accelerations and gravity. There is reason to believe that the body does not maintain perfect symmetry (equal masses) of the otoconia between the two inner ears, leading to slightly different sensitivities in vestibular sensing across the midline.^54^ The weightlessness of spaceflight made it clear that there is a central compensation mechanism for this tonic imbalance that is normally appropriate for an Earth-gravity environment; changes in ocular alignment (mediated by the otolith organs) and susceptibility to space motion sickness have been correlated with this asymmetry, which can be measured with simple assessment of the alignment of the two eyes in different gravity levels.^53^ Recognition of the role of such otolith asymmetry in vestibular-mediated responses has led to research that examines changes in one such response – vertical and torsional alignments of the eyes – under peripheral or central insult. Assessment of this response is aiding the understanding of traumatic injuries due to blast in military populations.^55^

## Conclusion

The challenges of performing physiological research in space should not be underestimated. This type of research is subject to many confounds: significant operational constraints, limited experimental controls, limited opportunities for repetition and validation, and small numbers of subjects. Despite these complications, NASA and its international partners have carried out a successful research program that has produced many important results. These results not only provide practical information to maintain astronaut health and safety in space,^56^ they also provide important information for fundamental scientific understanding. These scientific results also help us catalog the range of possible adaptive processes that might have aided in our development and adjustment to Earth, and suggest limits on the ability to live in extreme settings. All of these provide a broader perspective not just on physiology and human health, but on our place in the universe.

### Reporting summary

Further information on research design is available in the Nature Research Reporting Summary linked to this article.

## Supplementary information

Reporting Summary Checklist
